# Studies on Some Biologically Cobalt(II), Copper(II) and Zinc(II) Complexes With ONO, NNO and SNO Donor Pyrazinoylhydrazine-Derived Ligands

**DOI:** 10.1155/MBD.1998.267

**Published:** 1998

**Authors:** Zahid H. Chohan, Marapaka Praveen, Syed K. A. Sherazi

**Affiliations:** 1 Department of Chemistry Islamia University Bahawalpur Pakistan; 2 Department of Chemistry Washington University St. Louis MO 63130 USA

## Abstract

Biologically active complexes of Co(II), Ni(II), Cu(II) and Zn(II) with novel ONO, NNO and SNO donor pyrazinoylhydrazine-derived compounds have been prepared and characterized on the basis of analytical data and various physicochemical studies. Distorted octahedral structures for all the complexes have been proposed. The synthesized ligands and their complexes have been screened for their antibacterial activity against bacterial species *Escherichia coli*, *Pseudomonas aeruginosa*, *Staphylococcus aureus* and *Klebsiella pneumonae*. The activity data show the metal complexes to be more active than the parent free ligands against one or more bacterial species.


### STUDIES ON SOME BIOLOGICALLY COBALT(II), COPPER(II) AND ZINC(II) COMPLEXES WITH ONO, NNO AND SNO DONOR PYRAZINOYLHYDRAZINE-DERIVED LIGANDS

Zahid H. Chohan*[1], Marapaka Praveen[2] and Syed K. A. Sherazi[1]

[1] Department of Chemistry, Islamia University, Bahawalpur, Pakistan
[2] Department of Chemistry, Washington University, St. Louis 63130 MO, USA

### ABSTRACT

Biologically active complexes of Co(II), Ni(II), Cu(II) and Zn(II) with novel ONO, NNO and SNO donor pyrazinoylhydrazine-derived compounds have been prepared and characterized on the basis of analytical data and various physicochemical studies. Distorted octahedral structures for all the complexes have been proposed. The synthesized ligands and their complexes have been screened for their antibacterial activity against bacterial species *Escherichia coli, Pseudomonas aeruginosa, Staphylococcus aureus* and *Klebsiella pneumonae*. The activity data show the metal complexes to be more active than the parent free ligands against one or more bacterial species.

### INTRODUCTION

In recent years hydrazines and hydrazones have been actively investigated as donors because of their interesting biological properties[1-5] and varied ligational behaviour[6-9] towards different metal ions and manifestation of novel structural features in co-ordination chemistry. Much work has been done with their Schiff-base derived pyridine ring system[10]. Pyrazine system with an additional nitrogen ring becomes a weaker base[11], but it forms structurally important metal complexes[7-9]. The present investigation was undertaken to develop and study a novel variety of interactions of such Schiff-base ligands derived from the reaction of pyrazinoylhydrazine with furane-2-aldehyde, thiophene-2-aldehyde and pyrrol-2-aldehyde.
Many previous reports[12-15] and a part of our ongoing research program[16-20, 22,23] have indicated the role of metal ions in increasing the biological activity upon chelation.

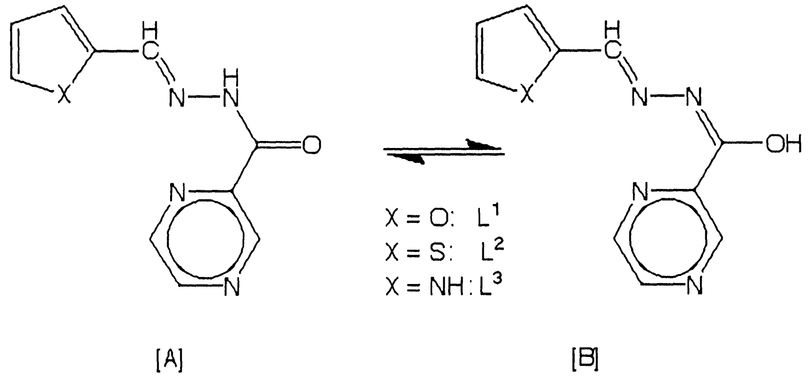

Figure 1 Structure of the ligand

In continuation to the same in the present studies we have synthesized novel pyrazinoylhydrazine derived tridentate ONO, NNO and SNO donor Schiff-base ligands L=$L^1$, $L^2$ and $L^3$ (Fig.1) and their metal complexes having a general formula [$(ML_2)Cl_2$] where M=Co(II), Ni(II), Cu(II) and Zn(I) and L=2-pyrazinoyl[1-(2-furanylmethylene)]hydrazide ($L^1$), 2-pyrazinoyl[1-(2-thienylmethylene)]hydrazide ($L^2$) and 2-pyrazinoyl[1-(2-pyrrolylmethylene)]hydrazide ($L^3$).
The synthesized Schiff-base ligands and their metal complexes have been screened for their possible antibacterial activity against bacterial strains of *Escherichia coli, Pseudomonas aeruginosa, Staphylococcus aureus* and *Klebsiella pneumonae* The antibacterial activity data of the ligands are shown to be substantially increased upon chelation against one or more bacterial strains.

### EXPERIMENTAL

### Material and Methods

All chemicals and solvents used were of Analar grade. All the metals were used as their metal(II) salts. IR, $^{1}H$ NMR and $^{13}C$ NMR spectra were recorded on Philips Analytical PU 9800 FTIR and Brucker 250 MHz instruments. $^{13}C$ resonances were assigned by the use of ref. 21. UV- Visible spectra were obtained on a Hitachi U-2000 double-beam spectrophotometer. Conductance of the metal complexes was determined in DMF on a YSI-32 model conductometer. Magnetic measurements were done on solid complexes using the Gouy method. Melting points were recorded on a Gallenkamp apparatus and are uncorrected. The antibacterial studies were carried out with the help of the Department of Pathology, Quaid-e-Azam Medical College, Bahawalpur, Pakistan.

### Preparation of Ligands

**2-Pyrazinoyl[1-(2-furanylmethylene)hydrazide] ($L^1$)**

Furan-2-aldehyde (0.8 mL, 0.96 g, 0.01 mol) in absolute ethanol (15 mL) was added to a magnetically stirred ethanol solution (25 mL) of pyrazinoylhydrazine (0.01 mol). Then 2-3 drops of concentrated sulfuric acid were added and mixture refluxed for 1 h. This mixture on cooling gave a solid product which was filtered, washed with ethanol (2x5 mL) then thoroughly with ether and dried. It was then crystallized in aqueous ethanol (50 %) to give ($L^1$) (0.92 g). The same methodology was adopted for the preparation of ($L^2$) (0.88 g) and ($L^3$) (0.94 g).

### Preparation of Metal Complexes

To a hot ethanolic solution (20 mL) of the ligand (0.02 mol) was added an aqueous solution (10 mL) of the respective metal (II) salt (0.01 mol). The mixture was refluxed for 2 h. The resulting mixture was cooled, filtered and reduced to nearly half of its volume. This concentrated solution was left overnight at room temperature which resulted in the formation of a solid product. The product thus obtained was filtered, washed with ethanol (2x10 mL) and dried. Crystallization from aqueous ethanol (50 %) gave the desired complexes (1-12).

### Antibacterial Studies

**Preparation of Discs.**

The ligand/complex (30 (g) in DMF (0.01mL) was applied on a paper disc, [prepared from blotting paper (3 mm diameter)] with the help of a micropipette. The discs were left in an incubator for 48 h at 37°C and then applied on the bacteria grown agar plates.

**Preparation of Agar Plates.**

Minimal agar was used for the growth of specific bacterial species. For the preparation of agar plates for *Escherichia coli*, MacConkey agar (50 g), obtained from Merck Chemical Company, was suspended in freshly distilled water (1 L). It was allowed to soak for 15 minutes and then boiled on a water bath until the agar was completely dissolved. The mixture was autoclaved for 15 minutes at 120° C and then poured into previously washed and sterilized Petri dishes and stored at 40°C for inoculation.

Table 1 Physical, Spectral and Analytical Data of the Ligands

| Schiff base/ Mol. Form. | M.P. (°C) | IR ($cm^{-1}$) | Calc (Found %) C | H | N |
|---|---|---|---|---|---|
| $L^1$ $C_{10}H_7N_4O_2$ [215.1] | 192 | 3275, 2915, 2865, 2015, 1945, 1714, 1620, 1350, 1060, 875, 345 | 55.83 (55.8) | 3.25 (3.0) | 26.03 (26.1) |
| $L^2$ $C_{10}H_7N_4OS$ [231.1] | 187 | 3275, 2910, 2865, 2015, 1945, 1714, 1620, 1345, 1060, 875, 345 | 51.96 (51.9) | 3.02 (3.3) | 24.23 (24.1) |
| $L^3$ $C_{10}H_8N_5O$ [214.1] | 179 | 3275, 2910, 2865, 2015, 1945, 1714, 1620, 1345, 1060, 877, 345 | 56.09 (56.2) | 3.73 (3.7) | 32.69 (32.8) |

**Procedure of Inoculation.**
Inoculation was done with the help of a platinum wire loop which was made red hot in a flame, cooled and then use for the application of bacterial strains.

**Application of Discs.**
A sterilized forceps was used for the application of paper disc on the already inoculated agar plates. When the discs were applied, they were incubated at 37° C for 24 h. The zone of inhibition was then measured (in diameter) around the disc.

### RESULTS AND DISCUSSION

The Schiff-base ligands $L^1$, $L^2$ and $L^3$ were prepared by a simple condensation reaction. The structural determination of these ligands was done with the help of IR, $^1$H-NMR, $^{13}$C-NMR and microanalytical data (Tables 1 & 2). The tentative assignment of some of the important infrared bands of ligands are recorded in Table 1. These bands in spectra of the free ligands show characteristic frequencies at 3275, 1714 and 1620 $cm^{-1}$. These are assigned to ν(NH), ν(C=O) and ν(C=N) stretches respectively.

Table 2 $^1$H NMR and $^{13}$C NMR Data of the Ligands

| L | $^1$HNMR (DMSO-$d_6$) (ppm) | $^{13}$CNMR (DMSO-$d_6$) (ppm) |
|---|---|---|
| $L^1$ | 4.54-4.56 (m, 1H, furanyl), 4.68 (dd, 1H, furanyl), 5.88 (s, 1H, furanyl), 6.13 (s, 1H, azomethine), 7.76-7.78 (m, 2H), 7.91-7.94(m, 1H), 8.34 (s, 1H, NH) | 104.71 ($C_3$), 112.24 ($C_7$), 121.36 ($C_5$), 123.22 ($C_4$), 124.1 ($C_6$), 126.2 ($C_2$), 129.12 ($C_8$), 137.27 ($C_9$), 152.31 (C=N), 187.38 (C=O). |
| $L^2$ | 4.55-4.57 (m, 1H, thienyl), 4.71 (dd, 1H, thienyl), 5.86(s,1H, thienyl), 6.15 (s, 1H, azomethine), 7.74 - 7.76 (m, 2H), 7.92-7.94 (m, 1H), 8.34 (s, 1H, NH) | 104.6 ($C_3$), 112.21 ($C_7$), 121.18 ($C_5$), 123.21 ($C_4$), 124.53 ($C_6$), 126.08 ($C_2$), 129.50 ($C_8$), 137.46 ($C_9$), 153.13 (C=N), 186.92 (C=O). |
| $L^3$ | 4.53-4.55 (m, 1H, pyrrolyl), 4.66 (dd, 1H, pyrrolyl), 5.85 (s, 1H, pyrrolyl), 6.15 (s, 1H, azomethine), 7.73-7.75 (m, 2H), 7.91-7.93 (m, 1H), 8.33 (s, 1H, NH), 8.48 (s, 1H, NH,), | 104.11 ($C_3$), 112.56 ($C_7$), 121.3 ($C_5$), 123.46 ($C_4$), 124.41 ($C_6$), 126.82 ($C_2$), 129.38 ($C_8$), 137.22 ($C_9$), 153.0 (C=N), 188.27 (C=O). |

The $^1$H-NMR spectra (Table 2) also display signals assignable to azomethine, amide and other expected ring protons. $^{13}$C-NMR spectra (Table 2) similarly, showed all carbons in their expected region. Also, the microanalytical data (Table 1) was found to be in agreement with the molecular structures of the title ligands. All the ligands apparently, contain a number of potential donor groups, which can act as co-ordination sites for metal chelation. Model studies as illustrated in Fig 1 show that these ligands act as tridentate and in no case they can exhibit a quadridentate behavior.

IR spectra of the ligands also show well defined ν(C=O) and ν(NH) modes and no stretching due to the presence of ν(OH) frequency in the region at 3360-3420 $cm^{-1}$ was found which was indicative of their more probable keto form (A) than the enol form (B). Furthermore, the absence of phenolic proton ν(OH) in $^1$H-NMR spectra confirmed keto configuration of the ligands.

All the metal complexes (1-12) were prepared by stoichiometric reactions of the respective metal(II) chlorides and respective ligands in the molar ratio M:L = 1:2. All complexes are air and moisture stable solids. They are soluble in DMSO, DMF and water and insoluble in other solvents. Molar conductance values of $10^{-3}$ M solutions of the complexes in DMSO (35-52 $ohm^{-1}$ $cm^2$ $mol^{-1}$) and water (75-87 $ohm^{-1}$ $cm^2$ $mol^{-1}$) indicated[24] the complexes to be non-electrolytes, although some solvolysis occurred.

**Magnetic Susceptibility**
The magnetic measurements (Table 3) for the solid complexes at room temperature are indicative of three unpaired electrons per Co(II) ion ($\mu_{eff}$ = 4.93-5.45 B.M), two unpaired electrons per Ni(II) ion ($\mu_{eff}$ = 2.87-3.22 B.M) and one unpaired electron per Cu(II) ion ($\mu_{eff}$ = 1.48-1.65 B.M) suggesting[25-28] octahedral geometry for Co(II) and Ni(II) and distorted octahedral geometry for Cu(II) complexes. The Zn(II) complexes were found to be diamagnetic.

Table 3 Physical Data of Metal Chelates

| No | Metal chelate/ Mol. Formula | Yied (%) | M.P (°C) | B.M ($\mu_{eff}$) | Calc (Found)% C H N |
|---|---|---|---|---|---|
| 1 | $[Co(L^1)_2(Cl)_2]$ $C_{20}H_{14}CoN_8O_4$ [560.03] | 79 | 226-228 | 4.93 | 42.89 2.49 19.99 (42.76) (2.58) (20.13) |
| 2 | $[Co(L^2)_2(Cl)_2]$ $C_{20}H_{14}CoN_8O_2S_2$ [592.03] | 87 | 221-222 | 5.45 | 40.57 2.36 18.91 (41.08) (2.88) (18.76) |
| 3 | $[Co(L^3)_2(Cl)_2]$ $C_{20}H_{16}CoN_{10}O_2$ [558.03] | 75 | 215-217 | 5.36 | 43.04 2.86 25.08 (42.85) (2.91) (25.48) |
| 4 | $[Ni(L^1)_2(Cl)_2]$ $C_{20}H_{14}NiN_8O_4$ [560.06] | 88 | 230-231 | 2.87 | 42.88 2.49 19.99 (42.56) (2.79) (20.24) |
| 5 | $[Ni(L^2)_2(Cl)_2]$ $C_{20}H_{14}NiN_8O_2S_2$ [589.76] | 88 | 233-235 | 3.22 | 40.57 2.36 18.91 (40.96) (2.51) (18.37) |
| 6 | $[Ni(L^3)_2(Cl)_2]$ $C_{20}H_{16}NiN_{10}O_2$ [558.06] | 75 | 220.222 | 3.18 | 43.04 2.86 25.08 (43.18) (2.77) (25.32) |
| 7 | $[Cu(L^1)_2(Cl)_2]C_{20}H_{14}CuN_8O_4$ [564.65] | 75 | 238-241 | 1.48 | 42.53 2.47 19.83 (42.64) (2.18) (20.09) |
| 8 | $[Cu(L^2)_2(Cl)_2]$ $C_{20}H_{14}CuN_8O_2S_2$ [596.65] | 83 | 231-233 | 1.65 | 40.25 2.34 18.77 (40.39) (2.51) (18.80) |
| 9 | $[Cu(L^3)_2(Cl)_2]$ $C_{20}H_{16}CuN_{10}O_2$ [584.92] | 87 | 228-230 | 1.56 | 42.69 2.84 24.88 (42.93) (2.57) (24.78) |
| 10 | $[Zn(L^1)_2(Cl)_2]$ $C_{20}H_{14}ZnN_8O_4$ [566.49] | 85 | 218-220 | dia | 42.40 2.47 19.77 (42.76) (2.81) (19.46) |
| 11 | $[Zn(L^2)_2(Cl)_2]$ $C_{20}H_{14}ZnN_8O_2S_2$ [598.49] | 87 | 211-214 | dia | 40.13 2.33 18.71 (40.55) (2.61) (18.47) |
| 12 | $[Zn(L^3)_2(Cl)_2]$ $C_{20}H_{16}ZnN_{10}O_2$ [564.49] | 88 | 202-204 | dia | 42.55 3.18 24.80 (42.78) (3.01) (24.93) |

Table 4 Spectral and Analytical Data of Metal Chelates

| No | IR ($cm^{-1}$) | $\lambda_{max}$ ($cm^{-1}$) |
|---|---|---|
| 1 | 3275 (NH), 1714 (C=O), 1615 (C=N), 510 (M-O), 360 (N-N) | 29205, 18352, 8165 |
| 2 | 3275 (NH), 1715 (C=O), 1612 (C=N), 510 (M-O), 365 (N-N) | 30265, 19225, 9740 |
| 3 | 3275 (NH), 1715 (C=O), 1615 (C=N), 510 (M-O), 367 (N-N) | 31260, 18550, 8595 |
| 4 | 3275 (NH), 1714 (C=O), 1612 (C=N), 510 (M-O), 365 (N-N) | 29671, 19185, 9690 |
| 5 | 3275 (NH), 1715 (C=O), 1615 (C=N), 445 (M-S), 365 (N-N) | 28950, 16220, 9455 |
| 6 | 3275 (NH), 1715 (C=O), 1610 (C=N), 450 (M-S), 365 (N-N) | 27375, 17523, 10112 |
| 7 | 3275 (NH), 1714 (C=O), 1612 (C=N), 445 (M-S), 367 (N-N) | 28567, 16832, 9875 |
| 8 | 3275 (NH), 1715 (C=O), 1610 (C=N), 445 (M-S), 365 (N-N) | 27845, 16555, 9742 |
| 9 | 3275 (NH), 1715 (C=O), 1615 (C=N), 385 (M-N), 365 (N-N) | 13965 |
| 10 | 3275 (NH), 1715 (C=O), 1615 (C=N), 395 (M-N), 367 (N-N) | 15220 |
| 11 | 3275 (NH), 1715 (C=O), 1612 (C=N), 390 (M-N), 365 (N-N) | 14867 |
| 12 | 3275 (NH), 1715 (C=O), 1612 (C=N), 388 (M-N), 365 (N-N) | 15111 |

**Infrared Spectra**

Co-ordination of the ligands with the metal ions was investigated by comparing the infrared spectra of the free ligands with the spectra of their metal complexes (Table 4). IR spectra of the ligands generally show bands at 3275 (s, sh), 1714 (s), 1620 (s) and 1000-1060 $cm^{-1}$ tentatively assigned to $\nu_{NH}$, $\nu_{C=O}$, $\nu_{C=N}$ and $\nu_{N-N}$ respectively[29,30]. In their metal complexes, the amide band remains at the same position as in the free ligand indicating that keto oxygen adjacent to this amide is not co-ordinated. Upward shift of the $\nu_{N-N}$ band and downward shift of azomethine linkage suggested[31,32] co-ordination through nitrogen of the azomethine group (-CH=N-). The pyrazine ring out of plane bending vibration at 345 $cm^{-1}$ moved to 367 $cm^{-1}$ indicating that the ring nitrogen is also involved in co-ordination.

Moreover, the new bands appearing in the spectra of the complexes and not observed in the spectra of the ligands within 510-525 $cm^{-1}$, 445-450 $cm^{-1}$ and 382-395 $cm^{-1}$ assigned[33,34] to M-O, M-S and M-N modes, respectively, indicated that the heteroatoms X (O, S or N) are also co-ordinated to the metal(II) ion.

Table 5 Antibacterial Activity Data

| Ligand/Chelate | M i c r | o b i a l | S p e c | c i e s |
|---|---|---|---|---|
| | a | b | c | d |
| $L^1$ | ++ | ++ | + | ++ |
| $L^2$ | + | ++ | ++ | + |
| $L^3$ | ++ | + | - | + |
| 1 | ++++ | +++ | +++ | ++++ |
| 2 | ++ | ++ | + | ++ |
| 3 | +++ | +++ | ++++ | +++ |
| 4 | +++ | ++ | +++ | ++ |
| 5 | +++ | ++++ | +++ | +++ |
| 6 | ++ | ++ | + | ++ |
| 7 | +++ | +++ | ++++ | +++ |
| 8 | +++ | +++ | +++ | ++ |
| 9 | ++++ | +++ | +++ | ++++ |
| 10 | + | ++ | + | ++ |
| 11 | +++ | +++ | ++ | +++ |
| 12 | ++ | ++ | +++ | ++ |

a=*Escherichia coli*, b=*Pseudomonas aeruginosa*,
c=*Staphylococcus aureus* d=*Klebsiella pneumonae*
Inhibition zone diameter (mm), +; 6-10, ++; 10-14, +++; 14-18, ++++; 18-22

**Electronic Spectra**

Electronic spectra of the metal complexes are recorded in Table 4. The spectra of the cobalt chelates show three bands observed at 8165-9740 $cm^{-1}$, 18352-19225 $cm^{-1}$ and 29205-32265 $cm^{-1}$ which may be assigned to ${}^{4}T_{1}g \rightarrow {}^{4}T_{2}g$ (F), ${}^{4}T_{1}g \rightarrow {}^{4}A_{2}g$ (F) and ${}^{4}T_{1}g \rightarrow {}^{4}T_{1}g$ (P) transitions, respectively, and are suggestive[35,36] of octahedral geometry around the cobalt ion. Three bands observed at 9455-10112 $cm^{-1}$, 16220-17523 $cm^{-1}$ and 27375-28950 $cm^{-1}$ in the spectra of the nickel(II) chelates are due to spin-allowed transitions from ${}^{3}A_{2}g \rightarrow {}^{3}T_{2}g$ (F), ${}^{3}A_{2}g \rightarrow {}^{3}A_{1}g$ (F) and ${}^{3}A_{2}g \rightarrow {}^{3}T_{1}g$ (P), transitions, respectively, in an octahedral environment[37,38]. The copper(II) chelates show only one band around 13965-15220 $cm^{-1}$ corresponding to ${}^{2}Eg \rightarrow {}^{2}T_{2}g$ transition, probably due to a distorted octahedral environment[39,40]

Based on the magnetic moment and spectral data, it may be tentatively proposed (Fig 2) that all the complexes possess an octahedral geometry in which all the ligands behaving as tridentate arrange themselves around the metal ion in such a way that a stable configuration of the metal chelate is attained.

**Antibacterial Studies**

The title ligands in comparison to their metal complexes were screened against bacterial species *Escherichia coli, Pseudomonas aeruginosa, Staphylococcus aureus and Klebsiella pneumonae and* in order to determine their antibacterial properties. The antibacterial activity was tested at a concentration 30μg/0/01 mL in DMF using paper disc diffusion method as reported earlier[41,42].

The results of these studies reported in Table 5 showed that the ligands and all their metal complexes are biologically active against one or more bacterial species and the metal complexes have been shown to be more antibacterial than the simple uncomplexed parent ligands.

**REFERENCES**

1. C. Pelizzi, G. Pelizzi and F. Vitali, *J. Chem. Soc (Dalton Trans)*., 1987, 177.
2. A. Mangia, C. Pelizzi and G. Pelizzi, *Acta. Crystallogr*., 1974, **30B**: 2146.
3. C. Pelizzi, G. Pelizzi, G. Predieri and S. Resola, *J. Chem. Soc (Dalton Trans)*., 1982, 1349.
4. M. B. Hursthouse, S. Amarasiri, A. Jayaweera and A. Quick, *J. Chem. Soc (DaltonTrans)*., 1979, 279.
5. R. Haran, J. Gairin and G. Commenges, *Inorg. Chim. Acta*, 1980, **46**: 63.
6. T. B. Murphy, D. K. Johnson, N. J. Rose, A. Aruffo and V. Schomaker, *Inorg. Chim. Acta*, 1982, **66**: L-67.
7. E. B. Flescher, D. Jeter and R. Flortan, *Inorg. Chem*., 1974, **13**: 1042.
8. E. B. Flescher and M. B. Lawson, *Inorg. Chem*., 1972, **11**: 2772.
9. C. L. Clein, E. D. Stevens, C. J. O'Connor, R. J. Majestes and L. M. Trefonas, *Inorg. Chim. Acta*, 1983, **70**: 151.
10. R. L. Dutta and M. M. Hossain, *J. Scient. Ind. Res*., 1985, **44**: 635.
11. A. Albert, R. Goldacre and J. Phillips, *J. Chem. Soc*., 1948, 2240.
12. D. R. Williams, "The Metals of Life-The Solution Chemistry of Metal Ions in Biological Systems", Van Nostrand, London, 1971.
13. S. Kirschner, Y. K. Wei, D. Francis and J. G. Bergman, *J. Med. Chem*., 1966, **9**: 369.
14. M. J. Clare, J. D. Heeschele, *Bioinorg. Chem*., 1973, **2**: 187.
15. B. Rosenberg and L. V. Camp, *Cancer. Res*., 1970, **30**: 1799.
16. Z. H. Chohan and A. Rauf, *Synth. React. Inorg. Met-Org. Chem*., 1996, **26**: 591.
17. Z. H. Chohan and A. Rauf, *J. Inorg. Biochem*., 1992, **46**: 41.
18. Z. H. Chohan and S. Kausar, *Chem. Pharm. Bull*., 1992, **40**: 2555.
19. Z. H. Chohan, M. Praveen and A. Ghaffar, *Metal-Based Drugs*, 1997, **4**: 267.
20. Z. H. Chohan and S. K. A. Sherazi, *Metal-Based Drugs*, 1997, **4**: 65, 69.
21. D. H. Williams and I. Fleming, "Spectroscopic Methods in Organic Chemistry", 4th Ed., McGraw Hill, London, 1989.
22. Z. H. Chohan and A. Rauf, *Metal-Based Drugs*, 1996, **3**: 211.
23. Z. H. Chohan and H. Pervez, *Synth. React. Inorg. Met-Org. Chem*.,1993, **23**: 1061.
24. W. J. Geary, *Coord. Chem. Rev*., 1971, **7**: 81.
25. R. H. Niswander, A. K. St Clair, S. R. Edmondson and L. T. Taylor, *Inorg. Chem*., 1975, **14**: 478.
26. R. C. Stoufer, D. W. Smith, E. A. Clevenger and T. E. Norris, *Inorg. Chem*., 1966, **5**: 1167.
27. M. Kato, H. B. Jonassen and J. C. Fanning, *Chem. Rev*., 1964, 64, 99.
28. C. J. Balhausen, "An Introduction to Ligand Field", McGraw Hill, New York, 1962.
29. K. Burger, I. Ruff and F. Ruff, *J. Inorg. Nucl. Chem*., 1965, 27, 179.
30. C. Pelizzi and G. Pelizzi, *Inorg. Chim. Acta*, 1976, 18, 39.
31. R. C. Aggarwal, N. K. Singh and R. P. Singh, *Inorg. Chem*., 1981, 20, 2794.
32. I. Gamo, *Bull. Chem. Soc. Japan*, 1961, 34, 1430.
33. L. J. Bellamy, "The Infrared Spectra of Complex Molecules", 3rd Ed, Methuen, London, 1966.
34. K. Nakamoto, "Infrared Spectra of Inorganic and Co-ordination Compounds", J. Wiley, New York, 2nd Ed, 1970.
35. A. D. Liehr, J. Phys. Chem., 1967, 67, 1314.
36. R. L. Carlin, "Transition Metal Chemistry", Ed. R. L. Carlin, Vol 1, Marcel Decker, New York, 1965.
37. A. B. P. Lever, "Inorganic Electronic Spectroscopy", Elsevier, Amsterdam, 1984.

38. D. W. Meek, R. S. Drago and T. S. Piper, Inorg. Chem., 1962, 1, 285.
39. W. E. Estes, D. P. Govel, W. E. Halfield and D. J. Hodgson, Inorg. Chem., 1978, 17, 1415.
40. A. B. P. Lever, J. Lewis and R. S. Nyholm, J. Chem. Soc., 1963, 2552.
41. Z. H. Chohan, Chem. Pharm. Bull., 1991, 39, 1578.
42. Z. H. Chohan and S. Kausar, Chem. Pharm. Bull., 1993, 41, 951.

**Received: May 28, 1998 - Accepted: June 26, 1998 -**
**Received in revised camera-ready format: July 2, 1998**

